# DRD3 Predicts Cognitive Impairment and Anxiety in Parkinson’s Disease: Susceptibility and Protective Effects

**DOI:** 10.3233/JPD-230292

**Published:** 2024-03-05

**Authors:** Alexandra Gonçalves, Alexandre Mendes, Joana Damásio, Nuno Vila-Chã, Daniela Boleixa, Bárbara Leal, Sara Cavaco

**Affiliations:** a Neuropsychology Service, Centro Hospitalar Universitário de Santo António, Porto, Portugal; b Faculty of Medicine, University of Porto, Porto, Portugal; c Neurology Department, Centro Hospitalar Universitário de Santo António, Porto, Portugal; d Unit for Multidisciplinary Research in Biomedicine (UMIB), Instituto de Ciências Biomédicas Abel Salazar (ICBAS), University of Porto, Porto, Portugal; e ITR – Laboratory for Integrative and Translational Research in Population Health, Porto, Portugal; f Departamento de Patologia e Imunologia Molecular, Immunogenetics Laboratory, Instituto de Ciências Biomédicas Abel Salazar (ICBAS), University of Porto, Porto, Portugal

**Keywords:** Parkinson’s disease, dopamine D3 receptor, cognition, anxiety

## Abstract

**Background:**

A possible genetic contribution of dopamine D3 receptor (DRD3) to cognitive impairment in Parkinson’s disease (PD) has yet to be investigated.

**Objective:**

To explore the effects of rs6280 (Ser9Gly) genotype on PD patients’ cognitive performance and to clarify possible interactions with psychopathology.

**Methods:**

Two hundred and fifty-three consecutive PD patients underwent neurological and neuropsychological evaluations, which included: Unified Parkinson’s Disease Rating Scale (UPDRS), Hoehn & Yahr scale (H&Y), Dementia Rating Scale-2 (DRS-2), and Hospital Anxiety and Depression Scale (HADS). rs6280 polymorphism was genotyped for all PD patients and for 270 ethnically matched healthy volunteers (HC). Non-parametric group comparisons and logistic regressions were used for data analyses.

**Results:**

rs6280 genotype did not differ between PD and HC groups. PD patients with rs6280 CC genotype had more impaired cognitive performance (i.e., <1st percentile of demographically adjusted norms) on DRS-2 subscales Initiation/Perseveration and Construction than those with TT genotype. These associations remained statistically significant when other covariates (e.g., demographic features, disease duration, severity of motor symptoms in OFF and ON states, anti-parkinsonian medication, and psychopathology symptoms) were taken into consideration. PD patients with rs6280 TC had less anxiety (i.e., HADS Anxiety≥11) than those with TT (*p* = 0.012). This association was also independent of other covariates.

**Conclusions:**

Study findings suggest that rs6280 CC genotype predisposes to executive dysfunction and visuoconstructional deficits, whereas the heterozygous genotype protects from anxiety in PD. These effects do not appear to be dependent of one another. rs6280 is not a genotypic susceptibility factor for PD.

## INTRODUCTION

Cognitive dysfunction is a common and debilitating nonmotor symptom of Parkinson’s disease (PD). The cognitive profile of patients with PD varies considerably, however cognitive deficits in the early stages of the disease (e.g., planning, initiation, monitoring of goal-directed behaviors, and working memory) are frequently associated with frontal–striatal dysfunction [1]. The conversion to dementia has been linked to posterior cortical dysfunction [2]. The neuropathological substrate of cognitive decline and dementia in PD has been a topic of great interest and debate in recent years.

DRD3, a G-protein couple receptor, is expressed mainly in neurons and glial cells throughout the central nervous system with marked expression in limbic areas. DRD3 modulates dopamine release and activation of signaling pathways through its autoreceptor function. The literature has provided evidence that DRD3 is involved in cognitive and social functions [3] and is implicated in mood disorders [4]. Studies in animal models suggest that when the D3 R function increases (e.g., through agonism, increased receptor expression), it impairs cognition and when D3 R function is reduced (e.g., through antagonism, lack of the receptor) it improves cognitive performance [3]. However, a neuropathological study in PD patients found that a reduction in DRD3 is related to pre-mortem presence of dementia and poor response to anti-parkinsonian medication [5].

The impact of DRD3 gene variation on measures of dopamine receptor binding is not yet completely understood. Though, it is known that DRD3 function can be modulated by the functional rs6280 polymorphism (25T > C), that leads to the missense substitution, of a serine for a glycine aminoacid in the N-terminal of DRD3 receptor (Ser9Gly). DRD3 predominantly acts as a presynaptic autoreceptor that inhibits dopamine release. The higher affinity of the glycine aminoacid (rs6280C allele) leads to an increased intracellular signaling and a decrease in extrasynaptic dopamine concentration under conditions of tonic dopamine release. The glycine autoreceptor may also predispose to reward-dependent elevated phasic dopamine signaling [6]. Clinically, the rs6280C allele in PD has been associated with visual hallucinations [7], impulse control disorders [8], and enhanced loss aversion in the absence of active impulse control disorder [9]. The rs6280C allele has also been associated with executive dysfunction in psychotic patients [10, 11] and substance abusers [12]. The poor cognitive functioning, specifically executive dysfunction, in first time psychotic episode carriers of the rs6280C allele is accompanied by lower grey matter volume in the hippocampus [11]. The rs6280C allele has also been linked to depression and anhedonia symptoms, including in PD patients [4]. This association was accompanied by increased resting-state activation (i.e., amplitude of low-frequency fluctuations) in the right medial frontal cortex [4].

These set of findings suggests that DRD3 receptor plays an important role in the intricate modulation of dopamine signaling pathways involved in the fine-tuning of cognitive functions and emotional processing. The main goals of the present study were to explore the effects of rs6280 genotype on cognitive test performance of PD patients and to clarify possible interactions with psychopathology symptoms. As observed in non-PD psychotic patients, we expected that PD patients with rs6280 CC and/or CT genotype would have poorer performance in executive functions than those with r6280 TT genotype.

## METHODS

### Subjects

Our cohort of PD patients has been previously described as part of a comprehensive observational study [13]. Briefly, from a series of 322 consecutive non-surgically treated PD subjects, only one patient refused to participate in the study. From this cohort (*n* = 321), 6 died prior to assessment (2 patients from respiratory infection, 1 from heart disease, and 3 from unknown cause), 2 were excluded because they developed other debilitating conditions, and 13 moved geographically to a region not dependent from our center or could not be reached between inclusion and assessment. For the present study, 47 patients were *a posteriori* excluded, due to illiteracy (i.e., had less than 3 years of education) (*n* = 27), the rs6280 genotyping could not be conducted (*n* = 16), patient’s inability to complete the Dementia Rating Scale-2 (DRS-2) (*n* = 4), and/or change in the diagnosis (*n* = 3). A total of 253 PD participants were included in the study.

A series of 270 ethnically matched volunteers, recruited from the community (i.e., healthy blood donors), integrated the healthy control (HC) group. HC subjects only participated in the genotyping component of the study.

All participants (or legal representatives) were informed about the nature of the study and gave their consent for participation, in accordance with the Declaration of Helsinki. The study was approved by local ethics committee.

### Procedures

#### Assessment protocol

Clinical records were reviewed to determine age at disease onset and to identify current medication. Age at the first motor symptom was considered age at PD onset. Current anti-parkinsonian medication was converted to levodopa equivalent dose (LED) [14]. Two movement disorder specialists (AM, NVC) evaluated patients’ motor symptoms after at least 12 h without anti-parkinsonian medication (OFF state) and under the effect of their habitual morning dose of medication (ON state), using the Unified Parkinson’s Disease Rating Scale (UPDRS) [15] and Hoehn & Yahr scale (H&Y) [16]. Each PD patient was classified as having tremor-dominant or non-tremor-dominant phenotype [17].

A trained neuropsychologist, blinded to the motor assessment scores, applied the Portuguese versions of DRS-2 [18] and Hospital Anxiety and Depression Scale (HADS) [19]. The DRS-2 is a measure of general cognitive status. It consists of 24 brief subtests whose scores are combined into five subscales of attention, initiation/perseveration, construction, conceptualization, and memory. DRS-2 total and subscales scores were adjusted for age and education, according to the national norms, and the first percentile of norms was used as cut-off for cognitive impairment. For those medically treated (98% of the patients), the neuropsychological tests were applied under the effect of their regular anti-parkinsonian medication (in ON state).

History of appendectomy was explored as a covariate, because a prior study with the same dataset revealed that history of appendectomy was a risk factor for cognitive impairment (i.e., DRS-2 Conceptualization and Memory subscales) in late onset PD [20]. History of appendectomy had been obtained *a posteriori* via phone contact, with the patient or a family member.

#### DNA extraction and genotyping

Peripheral blood samples were collected in 5% ethylenediaminetetraacetic acid (EDTA) tubes (VACUETTE^®^, Portugal). Genomic DNA was obtained from proteinase-K-treated peripheral blood leukocytes using a salting-out procedure as previously described [21]. The rs6280 polymorphism was genotyped using a pre-designed TaqMan allelic discrimination assay (C_949770_20; Thermo Fisher Scientific, USA) and NzySpeedy qPCR mastermix (Nzytech, Portugal) in a Rotor Gene 6000 RT-PCR machine (Qiagen, Germany).

### Statistical analyses

Descriptive statistics were used for group characterization. Chi-square test and Mann-Whitney test were applied to compare PD group with HC group. Multiple logistic regression analyses were used to explore group differences regarding rs6280 genotype, while taking into consideration sex as a covariate.

Chi-square test and Kruskal-Wallis test were applied to compare the demographic and clinical characteristics of PD patients according to rs6280 genotype. Simple and multiple logistic regressions were used to explore predictors of impairment in cognitive measures, other than rs6280. The following independent variables were included in the multiple logistic regression model: rs6280 genotype, sex, age, education, history of appendectomy, age at disease onset (≥55), disease duration (≤5 years, 6–10 years, and ≥11 years), levodopa equivalent dose, dopamine agonist, anti-depressant, UPDRS-II at OFF state, UPDRS-III at OFF and ON states, H&Y at OFF and ON states, non-tremor dominant, HADS Anxiety≥11, and HADS Depression≥11. Due to collinearity issues, UPDRS-III at ON state, and H&Y at OFF and ON states were not included in the initial regression model. In separate analyses, UPDRS-III OFF state was replaced by UPDRS-III ON, H&Y OFF, and H&Y ON in the regression model. Backward selection method was applied, with a threshold for variable removal of *p* > 0.100.

## RESULTS

**Table 1:** Characterization of the PD sample (*n* = 253). DRD3 genotype stratification

		Total Sample	rs6280			
		(*n* = 253)	TT (*n* = 117)	TC (*n* = 97)	CC (*n* = 39)	p
Sex	Male	136 (53.8%)	68 (58.1%)	48 (49.5%)	20 (51.3%)	0.427
Age		68 (62, 76)	69 (61, 77)	68 (63, 76)	67 (60, 73)	0.498
Education		4 (4, 8)	4 (4, 9)	4 (4, 6)	4 (4, 8)	**0.047**
History of appendectomy		28 (11.2%)	14 (12.0%)	8 (8.5%)	6 (15.8%)	0.460
Age at disease onset		60 (53, 69)	60 (51, 70)	60 (54, 69)	61 (51, 67)	0.668
Disease duration		6 (4, 11)	7 (4, 11)	6 (4, 12)	6 (5, 10)	0.976
Levodopa equivalent dose		740 (450, 1100)	740 (410, 1213)	720 (430, 1075)	800 (480, 1250)	0.724
Dopamine agonist		109 (43.1%)	44 (37.6%)	46 (47.4%)	19 (48.7%)	0.262
Anti-depressant		84 (33.2%)	38 (32.5%)	32 (33.0%)	14 (35.9%)	0.924
UPDRS-I	Total	2 (1, 4)	2 (1, 3)	2 (1, 4)	2 (1, 5)	0.974
	Item 3≥2	50 (19.8%)	20 (17.1%)	23 (23.7%)	7 (17.9%)	0.458
UPDRS-II	OFF	14 (8, 20)	13 (8, 20)	14 (9, 18)	16 (10, 22)	0.272
	ON	8 (4, 12)	7 (4, 12)	8 (4, 12)	8 (5, 12)	0.603
UPDRS-III	OFF	23 (24, 39)	31 (22, 39)	30 (24, 39)	32 (26, 38)	0.729
	ON	20 (15, 27)	20 (15, 26)	19 (14, 27)	22 (15, 28)	0.826
H&Y OFF	OFF	2.5 (2, 3)	2.5 (2, 3)	2.5 (2, 3)	2.5 (2, 3)	0.763
	ON	2 (2, 2.5)	2 (2, 2.5)	2 (2, 2.5)	2.3 (2, 2.5)	0.537
Non-tremor dominant		189 (76.9%)	87 (74.4%)	72 (74.2%)	30 (76.9%)	0.941
DRS-2	Total	40 (15.8%)	19 (16.2%)	13 (13.4%)	8 (20.5%)	0.581
	Attention	34 (13.4%)	15 (12.8%)	14 (14.4%)	5 (12.8%)	0.935
	Initiation/Perseveration	26 (10.3%)	9 (7.7%)	8 (8.2%)	9 (23.1%)	**0.016**
	Construction	27 (10.7%)	9 (7.7%)	8 (8.2%)	10 (25.6%)	**0.004**
	Conceptualization	18 (7.1%)	10 (8.5%)	3 (3.1%)	5 (12.8%)	0.097
	Memory	31 (12.3%)	15 (12.8%)	9 (9.3%)	7 (17.9%)	0.366
HADS	Anxiety≥11	45 (18.3%)	27 (23.5%	9 (9.8%)	9 (23.1%)	**0.028**
	Depression≥11	51 (20.7%)	21 (18.3%)	18 (19.6%)	12 (30.8%)	0.235

Missing data: history of appendectomy could not be determined in 4 patients; 5 patients were not medicated with anti-parkinsonian medication, therefore neither UPDRS-II, UPDRS-III nor H&Y were applied in ON; and HADS was not applied to 7 patients, due to patients’ inability to complete the questionnaire. DRS-2 scores are presented as frequency of impairment (i.e., performance < 1st percentile). Chi-square test and Kruskal-Wallis test was used for group comparisons.

### rs6280 in PD and HC

The PD group (*n* = 253) had a higher frequency of males (53.8% vs. 37.4%, *p* < 0.001) than the HC group (*n* = 270). However, the frequency of rs6280 TT, TC, and CC of the PD group (respectively, 46.2%, 38.3%, and 15.4%) was not statistically different (*p* = 0.279) from the HC group (respectively, 41.2%, 45.2%, and 13.6%). The allelic frequency (i.e., he relative frequency of an allele in a given group) of C in the PD cohort was 34.6% and in the HC group was 36.2%. The genotypes TT or TC were recorded in 84.6% of PD patients and in 86.4% of HC subjects (*p* = 0.555), whereas the genotypes TC or CC were recorded in 53.8% of PD patients and 58.8% of HC subjects (*p* = 0.242). These negative findings regarding rs6280 were confirmed by multiple logistic regression analyses, with sex as covariate.

### PD group characteristics

PD group (*n* = 253) was composed by 136 men (54%) and 117 women (46%). The group’s median (25th, 75th percentiles) age was 68 years (62, 76) and of education was 4 years (4, 8). A positive history of appendectomy was recorded in 11.2% of PD patients. The median age at disease onset was 60 years (53, 69) and disease duration at the time of the assessment was 6 years (4, 11). The median levodopa equivalent dose was 740 mg (450, 1100) and 43% of PD patients were taking dopamine agonists (94.5% were taking ropinirole and their median dose was 12 mg). Eighty-four patients (33.2%) were taking anti-depressant at the time of the assessment and 50 (19.8%) had a UPDRS-I item 3 score≥2. The median UPDRS-II, UPDRS-III, and H&Y scores OFF medication were respectively 14 (8, 20), 23 (24, 39), and 2.5 (2, 3). The median UPDRS-II, UPDRS-III and H&Y scores ON medication were respectively 8 (4, 12), 20 (15, 27), and 2 (2, 2.5). A non-tremor dominant phenotype was observed in 76.9% of PD patients. The frequencies of impaired performance on DRS-2 and of psychopathology symptoms on HADS are presented in Table 1.

### rs6280 and PD group demographic and clinical characteristics

As presented in Table 1 and 2, sex, age, history of appendectomy, age at disease onset, disease duration, levodopa equivalent dose, dopamine agonist, anti-depressant medication, UPDRS-I, UPDRS-I item 3 score≥2, UPDRS-II OFF and ON, UPDRS-III OFF and ON, and H&Y OFF and ON did not vary significantly with rs6280 genotype (*p* > 0.05). The median education for the three genotypes was 4 years.

### rs6280 and PD cognitive impairment

As presented in Table 1 and 2, PD patients with rs6280 CC genotype, in comparison to those with TT or TC genotypes, had more impaired cognitive performance (i.e., <1st percentile of demographically adjusted norms) on DRS-2 subscales Initiation/Perseveration (23.1% vs. 7.7% and 8.2% respectively, Chi-square test *p* = 0.016) and Construction (25.6% vs. 7.7% and 8.2% respectively, Chi-square test *p* = 0.004). No other significant association was found between rs6280 genotype and cognitive test performance, namely DRS-2 Total and subscales Attention, Conceptualization, and Memory.

rs6280 CC genotype remained associated with impaired performance on DRS-2 subscales Initiation/Perseveration (adjusted odds = 5.244; 95% CI: 1.400, 19.637; *p* = 0.014) and Construction (adjusted odds = 6.891; 95% CI: 2.087, 22.759; *p* = 0.002) in comparison to TT genotype, when sex, age, education, history of appendectomy, age at disease onset (≥55 years), disease duration (≤5 years, 6–10 years, and ≥11 years), levodopa equivalent dose, dopamine agonist intake, anti-depressant intake, UPDRS-II score OFF medication, UPDRS-III score OFF medication, non-tremor dominant, HADS Anxiety≥11, and HADS Depression > 11 were considered as covariates. The same pattern of results was observed for rs6280 genotype, when UPDRS-III OFF medication was replaced by UPDRS-III ON medication, H&Y OFF or H&Y ON in the regression model.

### Other predictors of cognitive impairment in PD

As shown in Table 2, simple logistic regressions revealed that higher scores on UPDRS-I, UDRS-II OFF and ON, UPDRS-III OFF and ON and H&Y OFF and ON were associated with impaired cognitive performance on DRS-2 Total and all subscales. Higher levodopa equivalent dose also increased the odds of impaired performance on DRS-2 Total and subscales Initiation/Perseveration and Memory. Older age, history of appendectomy, older age at disease onset, longer disease duration, UPDRS-I item 3 ≥ 2 and non-tremor dominant phenotype, and more severe symptoms of anxiety and depression were also related to more cognitive impairment on DRS-2 Total and/or subscales. Higher education and dopamine agonists were associated with lower odds of impairment on DRS-2 Total and/or subscales. No significant association was found between cognitive measures and anti-depressant intake.

**Table 2:** Predictors of cognitive impairment on DRS-2 Total and subscales. Simple logistic regressions analyses

	Total			Attention			Initiation/			Construction			Conceptualization			Memory		
						Perseveration											
		OR	95% CI	p	OR	95% CI	p	OR	95% CI	p	OR	95% CI	p	OR	95% CI	p	OR	95% CI	p
rs6280	TT
	TC	0.80	0.37, 1.12	0.563	1.15	0.52, 2.51	0.524	1.08	0.40, 2.91	0.881	1.08	0.40, 2.91	0.881	0.34	0.09, 1.28	0.111	0.70	0.29, 1.67	0.415
	CC	1.33	0.53, 3.34	0.542	1.00	0.34, 2.96	>0.999	3.60	1.31, 9.87	**0.013**	4.14	1.54, 11.13	**0.005**	1.57	0.50, 4.92	0.436	1.49	0.56, 3.97	0.428
Sex	Male	0.84	0.43, 1.64	0.604	0.73	0.36, 1.51	0.401	1.43	0.62, 3.28	0.403	0.56	0.25, 1.25	0.155	0.40	0.15, 1.11	0.079	0.78	0.37, 1.66	0.523
Age		1.10	1.05, 1.15	**<0.001**	1.02	0.98, 1.06	0.285	1.06	1.01, 1.10	**0.012**	1.08	1.03, 1.13	**0.001**	1.08	1.03, 1.14	**0.004**	1.07	1.02, 1.11	**0.002**
Education		0.86	0.75, 0.98	**0.024**	0.88	0.77, 1.01	0.070	0.91	0.79, 1.05	0.187	0.74	0.57, 0.95	**0.019**	0.89	0.75, 1.07	0.213	0.95	0.84, 1.06	0.335
History of appendectomy		1.55	0.59, 4.12	0.376	1.06	0.34, 3.27	0.918	2.31	0.79, 6.78	0.127	1.03	0.29, 3.69	0.960	3.79	1.23, 11.71	**0.021**	2.87	1.10, 7.48	**0.031**
Age at disease onset≥55 years		2.90	1.16, 7.21	**0.023**	0.79	0.37, 1.69	0.545	1.99	0.72, 5.49	0.183	2.10	0.76, 5.77	0.150	2.34	0.66, 8.34	0.189	0.93	0.41, 2.08	0.854
Disease duration	≤5 years
6–10 years		1.71	0.71, 4.12	0.233	0.91	0.36, 2.28	0.844	1.43	0.53, 3.88	0.484	1.25	0.45, 3.50	0.666	3.52	0.90, 13.72	0.070	2.36	0.76, 7.34	0.138
≥11 years		3.07	1.31, 7.20	**0.010**	1.77	0.76, 4.16	0.189	1.79	0.66, 4.90	0.256	2.27	0.86, 5.97	0.098	3.79	0.94, 15.20	0.060	6.47	2.26, 18.54	**<0.001**
Levodopa equivalent dose		1.00	1.00, 1.00	**0.043**	1.00	1.00, 1.00	0.130	1.00	1.00, 1.00	**0.020**	1.00	1.00, 1.00	0.270	1.00	1.00, 1.00	0.657	1.00	1.00, 1.00	**0.001**
Dopamine agonist		0.15	0.06, 0.40	**<0.001**	0.91	0.44, 1.90	0.809	0.28	0.10, 0.77	**0.014**	0.20	0.07, 0.60	**0.004**	0.15	0.03, 0.67	**0.013**	0.50	0.22, 1.13	0.096
Anti-depressant		1.82	0.92, 3.62	0.087	1.11	0.52, 2.38	0.781	1.85	0.81, 4.19	0.143	2.03	0.91, 4.54	0.086	1.67	0.64, 4.41	0.298	1.79	0.84, 3.83	0.135
UPDRS-I	Total	1.68	1.44, 1.95	**<0.001**	1.38	1.22, 1.56	**<0.001**	1.91	1.58, 2.31	**<0.001**	1.38	1.21, 1.56	**<0.001**	1.50	1.29, 1.75	**<0.001**	1.53	1.33, 1.76	**<0.001**
	Item 3≥2	3.05	1.46, 6.37	**0.003**	1.87	0.83, 4.21	0.133	6.19	2.65, 14.47	**<0.001**	2.74	1.17, 6.41	**0.021**	3.68	1.37, 9.87	**0.010**	3.06	1.37, 6.82	**0.006**
UPDRS-II	OFF	1.12	1.08, 1.17	**<0.001**	1.09	1.05, 1.14	**<0.001**	1.16	1.10, 1.22	**<0.001**	1.09	1.05, 1.14	**<0.001**	1.13	1.07, 1.20	**<0.001**	1.14	1.09, 1.20	**<0.001**
	ON	1.20	1.13, 1.27	**<0.001**	1.15	1.09, 1.21	**<0.001**	1.22	1.14, 1.31	**<0.001**	1.14	1.08, 1.20	**<0.001**	1.20	1.12, 1.28	**<0.001**	1.18	1.11, 1.25	**<0.001**
UPDRS-III	OFF	1.08	1.05, 1.12	**<0.001**	1.07	1.04, 1.11	**<0.001**	1.10	1.06, 1.14	**<0.001**	1.07	1.03, 1.10	**<0.001**	1.09	1.05, 1.14	**<0.001**	1.08	1.05, 1.12	**<0.001**
	ON	8.86	1.08, 1.18	**<0.001**	1.10	1.05, 1.14	**<0.001**	1.14	1.08, 1.20	**<0.001**	1.07	1.03, 1.12	**0.001**	1.11	1.06, 1.17	**<0.001**	1.09	1.05, 1.14	**<0.001**
H&Y	OFF	3.12	1.99, 4.90	**<0.001**	2.31	1.47, 3.61	**<0.001**	2.90	1.76, 4.76	**<0.001**	3.28	1.99, 5.42	**<0.001**	3.12	1.78, 5.48	**<0.001**	3.89	2.35, 6.44	**<0.001**
	ON	8.86	3.82, 20.54	**<0.001**	3.40	1.79, 6.46	**<0.001**	4.56	2.21, 9.44	**<0.001**	5.20	2.44, 11.11	**<0.001**	4.57	2.16, 9.71	**<0.001**	5.78	2.66, 12.54	**<0.001**
Non-tremor dominant		3.53	1.20, 10.34	**0.022**	1.68	0.66, 4.27	0.274	9.60	1.27, 72.38	**0.028**	1.55	0.56, 4.29	0.394	–	–	–	11.89	1.59, 89.04	**0.016**
HADS	Anxiety≥11	2.56	1.17, 5.60	**0.019**	3.48	1.55, 7.85	**0.003**	2.13	0.82, 5.53	0.120	2.20	0.89, 5.43	0.088	9.29	3.17, 27.19	**<0.001**	2.59	1.12, 5.60	**0.027**
	Depression≥11	2.83	1.33, 6.01	**0.007**	2.02	0.88, 4.62	0.095	3.42	1.40, 8.33	**0.007**	2.73	1.16, 6.45	**0.022**	4.35	1.55, 12.24	**0.005**	3.03	1.35, 6.79	**0.008**

DRS-2 subscales were recoded: >1st percentile –0 vs. ≤1st percentile –1. Appendectomy was coded: without past history–0 and with past history–1. Age at disease onset was recoded into: <55 years–0, ≥55 years–1. Disease duration was recoded into ≤5 years–0, 6–10 years–1, and ≥11 years–2. Non-tremor dominant was coded: no–0 and yes–1. HADS Anxiety and Depression were recoded into <11 –0 and ≥11 –1. Missing data: history of appendectomy could not be determined in 4 patients; 5 patients were not medicated with anti-parkinsonian medication, therefore neither UPDRS-II, UPDRS-III nor H&Y were applied in ON; and HADS was not applied to 7 patients, due to patients’ inability to complete the questionnaire. All patients with impaired DRS-2 Conceptualization subscale had non-tremor dominant phenotype.

**Table 3:** Predictors of anxiety and depression. Simple logistic regressions analyses

		Anxiety			Depression			UPDRS-1 item 3≥2		
		OR	95% CI	p	OR	95% CI	p	OR	95% CI	p
rs6280	TT
	TC	0.35	0.16, 0.80	**0.012**	1.09	0.54, 2.19	0.812	1.41	0.75, 2.64	0.281
	CC	0.96	0.41, 2.31	0.978	1.99	0.87, 4.56	0.104	1.33	0.57, 3.08	0.505
Sex	Male	0.49	0.25, 0.94	**0.033**	0.62	0.33, 1.16	0.133	0.28	0.15, 0.53	**<0.001**
Age		0.97	0.94, 1.00	**0.038**	1.03	1.00, 1.06	0.102	1.02	0.99, 1.05	0.137
Education		0.90	0.81, 1.00	0.059	0.88	0.79, 0.98	**0.024**	**0.86**	**0.77, 0.95**	**0.003**
History of appendectomy		0.79	0.26, 2.40	0.671	1.73	0.71, 4.23	0.226	2.29	1.03, 5.11	**0.042**
Age at disease onset≥55 years		0.38	0.20, 0.75	**0.005**	1.23	0.62, 2.44	0.550	0.57	0.31, 1.04	0.067
Disease duration	≤5 years
	6–10 years	2.09	0.92, 4.77	0.079	1.81	0.86, 3.81	0.120	2.20	1.05, 4.60	**0.037**
	≥11 years	2.83	1.23, 6.54	**0.015**	1.83	0.83, 4.02	0.133	2.96	1.42, 6.19	**0.004**
Levodopa equivalent dose		1.00	1.00, 1.00	0.142	1.00	1.00, 1.00	0.169	1.00	1.00, 1.00	**0.024**
Dopamine agonist		1.31	0.68, 2.50	0.420	0.58	0.31, 1.11	0.102	0.60	0.33, 1.10	0.099
Anti-depressant		1.64	0.84, 3.18	0.144	1.75	0.93, 3.29	0.083	3.72	1.96, 7.06	**<0.001**
UPDRS-I	Total	1.33	1.67, 1.53	**<0.001**	1.51	1.30, 1.74	**<0.001**	2.01	1.67, 2.42	**<0.001**
	Item 3≥2	2.39	1.14, 4.78	**0.021**	6.14	3.04, 12.40	**<0.001**	–
UPDRS-II	OFF	1.10	1.06, 1.15	**<0.001**	1.08	1.04, 1.13	**<0.001**	1.11	1.06, 1.15	**<0.001**
	ON	1.15	1.09, 1.22	**<0.001**	1.13	1.07, 1.20	**<0.001**	1.13	1.08, 1.19	**<0.001**
UPDRS-III	OFF	1.04	1.01, 1.08	**0.007**	1.06	1.03, 1.09	**<0.001**	1.05	1.03, 1.08	**<0.001**
	ON	1.06	1.02, 1.10	**0.005**	1.09	1.04, 1.13	**<0.001**	1.07	1.038, 1.11	**<0.001**
H&Y	OFF	1.73	1.12, 2.65	**0.008**	2.13	1.45, 3.39	**<0.001**	2.04	1.42, 2.93	**<0.001**
	ON	2.66	1.33, 4.92	**0.001**	3.18	1.82, 7.08	**<0.001**	2.42	1.46, 4.01	**<0.001**
Non-tremor dominant		1.43	0.65, 3.17	0.376	3.02	1.22, 7.48	**0.017**	5.82	2.03, 16.72	**0.001**
DRS-2	Total	2.48	1.17, 5.60	**0.018**	2.80	1.33, 6.01	**0.006**	3.26	1.74, 6.10	**<0.001**

HADS Anxiety and Depression were recoded into <11 –0 and ≥11 –1. Appendectomy was coded: without past history–0 and with past history–1. Age at disease onset was recoded into: <55 years–0, ≥55 years–1. Disease duration was recoded into ≤5 years–0, 6–10 years–1, and ≥11 years–2. Non-tremor dominant was coded: no–0 and yes–1. DRS-2 Total was recoded: >1st percentile –0 vs. ≤1st percentile –1. Missing data: HADS was not applied to 7 patients, due to patients’ inability to complete the questionnaire; history of appendectomy could not be determined in 4 patients; and 5 patients were not medicated with anti-parkinsonian medication, therefore neither UPDRS-II, UPDRS-III nor H&Y were applied in ON state.

### rs6280 and psychopathology in PD

As shown in Table 1, the frequency of anxiety varied according to rs6280 genotype. A simple logistic regression revealed that patients with rs6280TC genotype had less anxiety (i.e., HADS Anxiety subscale≥11) than those with the rs6280TT (9.8% vs. 23.5%; odds = 0.353, *p* = 0.012). This result remained statistically significant (adjusted odds = 0.242; 95% CI:0.096, 0.613; *p* = 0.003) even when other covariates as sex, age, education, history of appendectomy, age at disease onset (≥55 years), disease duration (≤5 years, 6–10 years, and ≥11 years), levodopa equivalent dose, dopamine agonist intake, anti-depressant, UPDRS-II score OFF medication, UPDRS-III score OFF medication, non-tremor dominant, and DRS-2 Total were taken into consideration. The same pattern of results was observed for rs6280 genotype, when UPDRS-III OFF medication was replaced by UPDRS-III score ON medication, H&Y OFF or H&Y ON in the regression model.As shown in Tables 1 and 3, the frequency of depression, as measured by HADS Depression subscale≥11, was not statistically different for rs6280 TT (18.3%), TC (19.6%), and CC (30.8%) genotypes (*p* = 0.235). Consistently, the frequency of UPDRS-I item 3 ≥ 2 did not vary significantly between genotypes: rs6280 TT (17.1%), TC (23.7%), and CC (17.9%) (*p* = 0.458).

### Other predictors of cognitive impairment and psychopathology in PD

As shown in Table 3, simple logistic regressions revealed that worse non-motor and motor experiences of daily living as measured by UPDRS-I and UPDRS-II OFF and ON medication, more severe symptoms on UPDRS-III OFF and ON medication, more advanced disease stage as measured by H&Y OFF and ON medication, and more cognitive difficulties on the DRS-2 were associated with more symptoms of anxiety and depression (i.e., HADS Anxiety≥11, and HADS Depression≥11). Female sex, younger age at assessment and disease onset, and longer disease duration were associated with more anxiety (i.e., HADS Anxiety≥11). Depression (i.e., HADS Depression≥11 and UPDRS-1 item 3 ≥ 2) was related to fewer years of education and was more common in PD patients with non-tremor dominant motor phenotype. UPDRS-1 item 3 ≥ 2, but not HADS Depression≥11, was associated with female sex, longer disease duration, and higher levodopa equivalent dose. No significant association was found with dopamine agonist.

## DISCUSSION

Study results revealed that PD patients with rs6280 CC genotype are more likely to have significant deficits on DRS-2 Initiation/Perseveration and Construction subscales [22]. The association between cognitive deficits and CC genotype remained statistically significant when demographic features, appendectomy history, disease duration, severity of non-motor and motor symptoms and disease stage (OFF and ON states), anti-parkinsonian medication, psychoactive medication, motor phenotype, and psychopathology symptoms were taken into consideration. These findings are consistent with the literature results from non-Parkinsonian psychotic patients [10, 11] and substance abusers [12]. Noteworthy, no association was found between rs6280 genotype and DRS-2 Total score in our cohort, suggesting that DRD3 polymorphism does not have a global impact on cognition in PD.

DRS-2 Initiation/Perseveration subscale explores the ability to initiate, maintain and terminate goal-directed behaviors through verbal (e.g., semantic fluency) and motor (e.g., copying alternating manual movements and graphomotor sequences) tasks, whereas the Construction subscale consists of copying simple designs. Notably, deficits in semantic fluency and copy of designs are known predictors of dementia in PD [23].

There are reports that global cognitive decline in PD, as measured by DRS-2 Total score, is accompanied by an Alzheimer’s disease pattern of brain atrophy (i.e., hippocampus and parietal–temporal cortex) [24]. There is some evidence that frontal lobe regions and the nucleus accumbens may be particularly involved in the performance of Initiation/Perseveration and Construction subscales in PD [25]. Dopamine D3 receptors are predominantly expressed in the nucleus accumbens [26], which is believed to regulate cognitive and socio-emotional processes by integrating information from limbic structures and the prefrontal cortex [27].

The increased impairment on certain cognitive tests by PD individuals with rs6280 CC genotype may reflect the higher affinity for dopamine, the increased dopamine-mediated cyclic adenosine monophosphate (cAMP) response, and the prolonged mitogen-associated protein kinase (MAPK) signal characteristic of the Gly variant [28]. The presence of the Gly aminoacid attenuates the function of the D3 receptor [29]. An inverted-U curve between dopaminergic signaling and cognition has been hypothesized [30, 31], where too little or too much dopaminergic signaling impairs cognitive performance. In the present study, almost all patients were ON medication at cognitive assessment. Dopaminergic drugs may produce variable effects on cognitive performance, depending on the baseline levels of dopamine in certain brain regions, which may partly reflect genetic predisposition, and on the specific cognitive processes involved [30, 31]. Increasing levels of dopamine may benefit performance on some cognitive tasks and have a detrimental effect on others [30].

In the present study, PD patients taking dopamine agonist had better cognitive performance than those without dopamine agonist. An important confounder is that, in clinical practice, dopamine agonists are usually administered in monotherapy or adjunct therapy to younger patients in the early or stable PD [32], which is consistently related to more intact cognition in PD. Nonetheless, the significant association between dopamine agonist and cognition remained statistically significant, even when other covariates were taken into account. The preferred dopamine agonist in our cohort was ropinirole, a non-ergoline dopamine agonist with high D2/D3 affinity. The limited evidence available suggests that ropinirole may have relatively small adverse effects on cognition [33–35]. Ropinirole has been shown to selectively reduce proactive inhibition (i.e., slowing of motor activity in anticipation of stopping), but not reactive inhibition (i.e., response to sudden sensory cues and serves to abruptly stop motor activity) in healthy adults [36]. A reduction in proactive inhibition could potentially favor semantic fluency in PD patients [37]. There is evidence that pramipexole, another agonist with D3 affinity used in our cohort, may have negative effects on multiple cognitive functions, including semantic fluency [33, 38].

In our cohort, a screening measure detected anxiety in 18% and depression in 21% of PD patients. These frequencies are roughly similar to those reported by a previous study that also used screening tools and applied conservative cut-offs [39]. Though, these figures are lower than the estimated prevalence of clinically significant anxiety and depression symptoms reported by meta-analyses [40, 41]. Consistent with the literature, anxiety in PD patients was associated with female sex, younger age at disease onset and at assessment, and more severe motor symptoms [39, 42]. Depression was also associated with higher UPDRS-II, UPDRS-III, and H&Y scores [43]. Study findings provided further support to the notion that lower education and poorer cognitive performance are associated with anxiety and depression in PD patients [43]. No significant effect of levodopa equivalent dose or dopamine agonist was observed on psychopathology symptoms. Though, PD patients taking dopamine agonists tended to have lower odds of depression (i.e., HADS Depression≥11 and UPDRS-1 item 3 ≥ 2). Dopamine agonists are currently recommended to treat depression in PD [44].

PD patients with the rs6280 TC genotype had lower risk of anxiety than patients with the rs6280TT genotype. Another study explored the relationship between rs6280 and anxiety symptoms in PD and reported no significant association between the C allele and symptoms of anxiety [45]. Anxiety and depression are frequently comorbid in PD [46]. Theoretically, anxiety is associated with a state of physiological hyperarousal, whereas symptoms of anhedonia and the absence of positive affect are specific to depression [47, 48]. The pathophysiology of PD-related anxiety is complex and remains to be elucidated [46, 49]. Though, it is reasonable to speculate that rs6280 TC genotype may directly or indirectly protect from anxiety in PD patients treated with anti-parkinsonian medication.

The rs6280 was not statistically related to depression symptoms (i.e., HADS Depression≥11 and UPDRS-1 item 3 ≥ 2) nor with anti-depressant medication. Though, there is evidence in the literature that rs6280 polymorphism may be implicated in the pathogenesis of depression. A study found that PD patients with the rs6280C allele had more severe depression symptoms (i.e., with predominant anhedonia) than those without the rs6280C allele. Moreover, these depression symptoms were positively correlated with activation of the right medial frontal gyrus [4].

DRD3 genotypic composition was not statistically different in our cohort of PD patients and a population-based HC group, supporting the null hypothesis regarding the rs6280 polymorphism and the risk of PD [50, 51]. Though, there are reports in the literature that rs6280 CC genotype maybe more frequent in PD patients [8].

The consecutive recruitment of non-surgically treated PD patients, the neurological evaluation in both OFF and ON states by movement disorders specialists, and the cognitive assessment in ON state conducted by a neuropsychologist blinded to the neurological and the genetic results are strengths of the study. DRS-2 was completed by most patients of the cohort (including bedridden and/or with very low cognitive functioning), which reduces the risk of sample bias. As a single-center study, the generalizability of the research findings is limited. The replication of the study in other populations is necessary. Nevertheless, the rs6280 allelic frequency found in our population (allele *C* = 34.6%) is aligned with those reported for the European population (https://www.ncbi.nlm.nih.gov/snp/rs6280#frequency_tab) [52] The present research focused on the effects of the rs6280 genotype on PD patients’ cognitive test performance, while taking into consideration other known predictors (e.g., demographic characteristics, appendectomy history, motor symptoms, dopaminergic treatment, psychoactive medication, and psychopathology symptoms). However, the list of potential covariates was not exhaustive. For instance, other polymorphisms (e.g., COMT158, APOE, E326K) that are known to affect the cognitive trajectory of patients with idiopathic PD [53, 54] were not explored.

The cognitive evaluation was limited to the DRS-2, a global scale commonly used both in clinical practice and research. DRS-2 scores (both Total and subscales) were adjusted to the demographic characteristics of the individuals based on norms and a conservative cut-off was applied. This approach limits the risk of overestimating cognitive impairment, but also increases the insensitivity to milder cognitive deficits. DRS-2 is currently recommended by Movement Disorders Society to evaluate global cognitive performance in PD patients, because it is considered reliable, valid, and sensitive to change in PD and evaluates cognitive domains commonly affected in PD, such as executive abilities, attention, visuospatial skills, and memory [55]. As a global assessment scale, ceiling effects may occur. However, it is considered a clinically valid measure to differentiate levels of cognitive impairment in demented patients [56]. DRS-2 subscales are generally considered valid measures of the respective constructs [22, 57–61]. Though, the reported indicators of construct validity are relatively modest, thus probably reflecting the non-specificity to a single cognitive domain for each subscale and the screening nature of the tests. The interpretation of the study findings is also limited by the cross-sectional nature of the study. A longitudinal design may clarify the cognitive trajectories of PD patients according to their rs6280 genotype.

In conclusion, PD patients with rs6280 CC genotype are more likely to have deficits on measures of executive and visuoconstructional functions than those with TT genotype, suggesting a genetic predisposition to the development of impairment in cognitive domains related to the frontal lobes. The current study also revealed that rs6280 TC genotype may play a protective role on anxiety, but not on depression. These novel findings ought to be confirmed in other PD cohorts.

## Data Availability

The data supporting the findings of this study are available on request from the corresponding author. The data are not publicly available due to privacy or ethical restrictions.

## References

[1] Pagonabarraga J , Kulisevsky J (2012) Cognitive impairment and dementia in Parkinson’s disease. Neurobiol Dis 46, 590–596. 22484304 10.1016/j.nbd.2012.03.029

[2] Williams-Gray CH , Evans JR , Goris A , Foltynie T , Ban M , Robbins TW , Brayne C , Kolachana BS , Weinberger DR , Sawcer SJ , Barker RA (2009) The distinct cognitive syndromes of Parkinson’s disease: 5 year follow-up of the CamPaIGN cohort. Brain 132, 2958–2969. 19812213 10.1093/brain/awp245

[3] Kiss B , Laszlovszky I , Kramos B , Visegrady A , Bobok A , Levay G , Lendvai B , Roman V (2021) Neuronal dopamine D3 receptors: Translational implications for preclinical research and CNS disorders. Biomolecules 11, 104. 33466844 10.3390/biom11010104 PMC7830622

[4] Zhi Y , Yuan Y , Si Q , Wang M , Shen Y , Wang L , Zhang H , Zhang K (2019) The association between DRD3 Ser9Gly polymorphism and depression severity in Parkinson’s disease. Parkinsons Dis 2019, 1642087. 31143436 10.1155/2019/1642087 PMC6501220

[5] Joyce JN , Ryoo H , Gurevich EV , Adler C , Beach T (2001) Ventral striatal D(3) receptors and Parkinson’s disease. Parkinsonism Relat Disord 7, 225–230. 11331190 10.1016/s1353-8020(00)00060-2

[6] Savitz J , Hodgkinson CA , Martin-Soelch C , Shen PH , Szczepanik J , Nugent A , Herscovitch P , Grace AA , Goldman D , Drevets WC (2013) The functional DRD3 Ser9Gly polymorphism (rs6280) is pleiotropic, affecting reward as well as movement. PLoS One 8, e54108. 23365649 10.1371/journal.pone.0054108 PMC3554713

[7] Redensek S , Flisar D , Kojovic M , Gregoric Kramberger M , Georgiev D , Pirtosek Z , Trost M , Dolzan V (2019) Dopaminergic pathway genes influence adverse events related to dopaminergic treatment in Parkinson’s disease. Front Pharmacol 10, 8. 30745869 10.3389/fphar.2019.00008 PMC6360186

[8] Krishnamoorthy S , Rajan R , Banerjee M , Kumar H , Sarma G , Krishnan S , Sarma S , Kishore A (2016) Dopamine D3 receptor Ser9Gly variant is associated with impulse control disorders in Parkinson’s disease patients. Parkinsonism Relat Disord 30, 13–17. 27325396 10.1016/j.parkreldis.2016.06.005

[9] Rajan R , Krishnan S , Sarma G , Sarma SP , Kishore A (2018) Dopamine receptor D3 rs6280 is associated with aberrant decision-making in Parkinson’s disease. Mov Disord Clin Pract 5, 413–416. 30363458 10.1002/mdc3.12631 PMC6174438

[10] Bombin I , Arango C , Mayoral M , Castro-Fornieles J , Gonzalez-Pinto A , Gonzalez-Gomez C , Moreno D , Parellada M , Baeza I , Graell M , Otero S , Saiz PA , Patino-Garcia A (2008) DRD3, but not COMT or DRD2, genotype affects executive functions in healthy and first-episode psychosis adolescents. Am J Med Genet B Neuropsychiatr Genet 147B, 873–879. 18351593 10.1002/ajmg.b.30710

[11] Kang Y , Zhang Y , Huang K , Wang Z (2023) The genetic influence of the DRD3 rs6280 polymorphism (Ser9Gly) on functional connectivity and gray matter volume of the hippocampus in patients with first-episode, drug-naive schizophrenia. Behav Brain Res 437, 114124. 36154848 10.1016/j.bbr.2022.114124

[12] Limosin F , Romo L , Batel P , Ades J , Boni C , Gorwood P (2005) Association between dopamine receptor D3 gene BalI polymorphism and cognitive impulsiveness in alcohol-dependent men. Eur Psychiatry 20, 304–306. 15935433 10.1016/j.eurpsy.2005.02.004

[13] Mendes A , Goncalves A , Vila-Cha N , Moreira I , Fernandes J , Damasio J , Teixeira-Pinto A , Taipa R , Lima AB , Cavaco S (2015) Appendectomy may delay Parkinson’s disease onset. Mov Disord 30, 1404–1407. 26228745 10.1002/mds.26311

[14] Tomlinson CL , Stowe R , Patel S , Rick C , Gray R , Clarke CE (2010) Systematic review of levodopa dose equivalency reporting in Parkinson’s disease. Mov Disord 25, 2649–2653. 21069833 10.1002/mds.23429

[15] Fahn S , Elton R Members of the UPDRS Development Committee (1987) Unified Parkinson’s Disease Rating Scale. In Recent Developments in Parkinson’s Disease, Vol. 2, Fahn S, Marsden CD, Calne DB, Goldstein M, eds. Macmillan Healthcare Information, Florham Park, NJ, pp. 153-163, 293-304.

[16] Hoehn MM , Yahr MD (1967) Parkinsonism: Onset, progression and mortality. Neurology 17, 427–442. 6067254 10.1212/wnl.17.5.427

[17] Jankovic J , McDermott M , Carter J , Gauthier S , Goetz C , Golbe L , Huber S , Koller W , Olanow C , Shoulson I , et al. (1990) Variable expression of Parkinson’s disease: A base-line analysis of the DATATOP cohort. The Parkinson Study Group. Neurology 40, 1529–1534. 2215943 10.1212/wnl.40.10.1529

[18] Cavaco S , Teixeira-Pinto A (2011) Manual da Versão Portuguesa da Dementia Rating Scale-2, CEGOC-TEA, Lisboa.

[19] Pais-Ribeiro J , Silva I , Ferreira T , Martins A , Meneses R , Baltar M (2007) Validation study of a Portuguese version of the Hospital Anxiety and Depression Scale . Psychol Health Med 12, 225–235quiz 235-227. 17365902 10.1080/13548500500524088

[20] Goncalves AR , Mendes A , Vila-Cha N , Damasio J , Fernandes J , Cavaco SM (2021) Past appendectomy may be related to early cognitive dysfunction in Parkinson’s disease. Neurol Sci 42, 123–130. 32529319 10.1007/s10072-020-04507-1

[21] Miller SA , Dykes DD , Polesky HF (1988) A simple salting out procedure for extracting DNA from human nucleated cells. Nucleic Acids Res 16, 1215. 3344216 10.1093/nar/16.3.1215 PMC334765

[22] Lopez FV , Kenney LE , Ratajska A , Jacobson CE , Bowers D (2023) What does the Dementia Rating Scale-2 measure? The relationship of neuropsychological measures to DRS-2 total and subscale scores in non-demented individuals with Parkinson’s disease. Clin Neuropsychol 37, 174–193. 34779350 10.1080/13854046.2021.1999505 PMC9107526

[23] Weil RS , Schrag AE , Warren JD , Crutch SJ , Lees AJ , Morris HR (2016) Visual dysfunction in Parkinson’s disease. Brain 139, 2827–2843. 27412389 10.1093/brain/aww175 PMC5091042

[24] Weintraub D , Dietz N , Duda JE , Wolk DA , Doshi J , Xie SX , Davatzikos C , Clark CM , Siderowf A (2012) Alzheimer’s disease pattern of brain atrophy predicts cognitive decline in Parkinson’s disease. Brain 135, 170–180. 22108576 10.1093/brain/awr277 PMC3316476

[25] Planche V , Munsch F , Pereira B , de Schlichting E , Vidal T , Coste J , Morand D , de Chazeron I , Derost P , Debilly B , Llorca PM , Lemaire JJ , Marques A , Durif F (2018) Anatomical predictors of cognitive decline after subthalamic stimulation in Parkinson’s disease. Brain Struct Funct 223, 3063–3072. 29736590 10.1007/s00429-018-1677-2

[26] Larson ER , Ariano MA (1995) D3 and D2 dopamine receptors: Visualization of cellular expression patterns in motor and limbic structures. Synapse 20, 325–337. 7482292 10.1002/syn.890200406

[27] Goto Y , Grace AA (2008) Limbic and cortical information processing in the nucleus accumbens. Trends Neurosci 31, 552–558. 18786735 10.1016/j.tins.2008.08.002 PMC2884964

[28] Jeanneteau F , Funalot B , Jankovic J , Deng H , Lagarde JP , Lucotte G , Sokoloff P (2006) A functional variant of the dopamine D3 receptor is associated with risk and age-at-onset of essential tremor. Proc Natl Acad Sci U S A 103, 10753–10758. 16809426 10.1073/pnas.0508189103 PMC1502303

[29] Liu YZ , Tang BS , Yan XX , Liu J , Ouyang DS , Nie LN , Fan L , Li Z , Ji W , Hu DL , Wang D , Zhou HH (2009) Association of the DRD2 and DRD3 polymorphisms with response to pramipexole in Parkinson’s disease patients. Eur J Clin Pharmacol 65, 679–683. 19396436 10.1007/s00228-009-0658-z

[30] Cools R , D’Esposito M (2011) Inverted-U-shaped dopamine actions on human working memory and cognitive control. Biol Psychiatry 69, e113–e125. 21531388 10.1016/j.biopsych.2011.03.028 PMC3111448

[31] Cools R , Barker RA , Sahakian BJ , Robbins TW (2001) Enhanced or impaired cognitive function in Parkinson’s disease as a function of dopaminergic medication and task demands. Cereb Cortex 11, 1136–1143. 11709484 10.1093/cercor/11.12.1136

[32] Fox SH , Katzenschlager R , Lim SY , Barton B , de Bie RMA , Seppi K , Coelho M , Sampaio C , Movement Disorder Society Evidence-Based Medicine C (2018) International Parkinson and movement disorder society evidence-based medicine review: Update on treatments for the motor symptoms of Parkinson’s disease. Mov Disord 33, 1248–1266. 29570866 10.1002/mds.27372

[33] Shepherd TA , Edelstyn NMJ , Longshaw L , Sim J , Watts K , Mayes AR , Murray M , Ellis SJ (2018) Feasibility of a randomized single-blind crossover trial to assess the effects of the second-generation slow-release dopamine agonists pramipexole and ropinirole on cued recall memory in idiopathic mild or moderate Parkinson’s disease without cognitive impairment. Pilot Feasibility Stud 4, 11. 28694990 10.1186/s40814-017-0154-7 PMC5501424

[34] Zagmutt FJ , Tarrants ML (2012) Indirect comparisons of adverse events and dropout rates in early Parkinson’s disease trials of pramipexole, ropinirole, and rasagiline. Int J Neurosci 122, 345–353. 22304415 10.3109/00207454.2012.660586

[35] Mavrikaki M , Schintu N , Nomikos GG , Panagis G , Svenningsson P (2014) Ropinirole regulates emotionality and neuronal activity markers in the limbic forebrain. Int J Neuropsychopharmacol 17, 1981–1993. 24852388 10.1017/S1461145714000728

[36] Rawji V , Rocchi L , Foltynie T , Rothwell JC , Jahanshahi M (2020) Ropinirole, a dopamine agonist with high D(3) affinity, reduces proactive inhibition: A double-blind, placebo-controlled study in healthy adults. Neuropharmacology 179, 108278. 32827517 10.1016/j.neuropharm.2020.108278 PMC7575901

[37] Tiedt HO , Ehlen F , Klostermann F (2022) Dopamine-related reduction of semantic spreading activation in patients with Parkinson’s disease. Front Hum Neurosci 16, 837122. 35431839 10.3389/fnhum.2022.837122 PMC9008217

[38] Brusa L , Bassi A , Stefani A , Pierantozzi M , Peppe A , Caramia MD , Boffa L , Ruggieri S , Stanzione P (2003) Pramipexole in comparison to l-dopa: A neuropsychological study. J Neural Transm (Vienna) 110, 373–380. 12658365 10.1007/s00702-002-0811-7

[39] Zhu K , van Hilten JJ , Marinus J (2017) Onset and evolution of anxiety in Parkinson’s disease. Eur J Neurol 24, 404–411. 28032408 10.1111/ene.13217

[40] Broen MP , Narayen NE , Kuijf ML , Dissanayaka NN , Leentjens AF (2016) Prevalence of anxiety in Parkinson’s disease: A systematic review and meta-analysis. Mov Disord 31, 1125–1133. 27125963 10.1002/mds.26643

[41] Reijnders JS , Ehrt U , Weber WE , Aarsland D , Leentjens AF (2008) A systematic review of prevalence studies of depression in Parkinson’s disease . Mov Disord 23, 183–189.quiz 313 17987654 10.1002/mds.21803

[42] Brown RG , Landau S , Hindle JV , Playfer J , Samuel M , Wilson KC , Hurt CS , Anderson RJ , Carnell J , Dickinson L , Gibson G , van Schaick R , Sellwood K , Thomas BA , Burn DJ , Group P-PS (2011) Depression and anxiety related subtypes in Parkinson’s disease. J Neurol Neurosurg Psychiatry 82, 803–809. 21217155 10.1136/jnnp.2010.213652

[43] Cong S , Xiang C , Zhang S , Zhang T , Wang H , Cong S (2022) Prevalence and clinical aspects of depression in Parkinson’s disease: A systematic review and meta-analysis of 129 studies. Neurosci Biobehav Rev 141, 104749. 35750224 10.1016/j.neubiorev.2022.104749

[44] Aguera-Ortiz L , Garcia-Ramos R , Grandas Perez FJ , Lopez-Alvarez J , Montes Rodriguez JM , Olazaran Rodriguez FJ , Olivera Pueyo J , Pelegrin Valero C , Porta-Etessam J (2021) Focus on depression in Parkinson’s disease: A Delphi consensus of experts in psychiatry, neurology, and geriatrics. Parkinsons Dis 2021, 6621991. 33628415 10.1155/2021/6621991 PMC7884180

[45] Eryilmaz IE , Erer S , Zarifoglu M , Egeli U , Karakus E , Yurdacan B , Cecener G , Tunca B , Colakoglu B , Bora Tokcaer A , Saka E , Demirkiran M , Akbostanci C , Dogu O , Kaleagasi H , Kenangil G , Cakmur R , Elibol B (2020) Contribution of functional dopamine D2 and D3 receptor variants to motor and non-motor symptoms of early onset Parkinson’s disease. Clin Neurol Neurosurg 199, 106257. 33039854 10.1016/j.clineuro.2020.106257

[46] Dan R , Ruzicka F , Bezdicek O , Ruzicka E , Roth J , Vymazal J , Goelman G , Jech R (2017) Separate neural representations of depression, anxiety and apathy in Parkinson’s disease. Sci Rep 7, 12164. 28939804 10.1038/s41598-017-12457-6 PMC5610322

[47] Clark LA , Watson D (1991) Tripartite model of anxiety and depression: Psychometric evidence and taxonomic implications. J Abnorm Psychol 100, 316–336. 1918611 10.1037//0021-843x.100.3.316

[48] Craske MG , Rauch SL , Ursano R , Prenoveau J , Pine DS , Zinbarg RE (2009) What is an anxiety disorder? Depress Anxiety 26, 1066–1085. 19957279 10.1002/da.20633

[49] Pecina M , Sikora M , Avery ET , Heffernan J , Pecina S , Mickey BJ , Zubieta JK (2017) Striatal dopamine D2/3 receptor-mediated neurotransmission in major depression: Implications for anhedonia, anxiety and treatment response. Eur Neuropsychopharmacol 27, 977–986. 28870407 10.1016/j.euroneuro.2017.08.427 PMC5623119

[50] Keeling BH , Vilarino-Guell C , Ross OA , Wszolek ZK , Uitti RJ , Farrer MJ (2009) DRD3 Ser9Gly and HS1BP3 Ala265Gly are not associated with Parkinson disease. Neurosci Lett 461, 74–75. 19524641 10.1016/j.neulet.2009.05.084 PMC2757072

[51] McGuire V , Van Den Eeden SK , Tanner CM , Kamel F , Umbach DM , Marder K , Mayeux R , Ritz B , Ross GW , Petrovitch H , Topol B , Popat RA , Costello S , Manthripragada AD , Southwick A , Myers RM , Nelson LM (2011) Association of DRD2 and DRD3 polymorphisms with Parkinson’s disease in a multiethnic consortium. J Neurol Sci 307, 22–29. 21663922 10.1016/j.jns.2011.05.031 PMC3155471

[52] Sherry ST , Ward MH , Kholodov M , Baker J , Phan L , Smigielski EM , Sirotkin K (2001) dbSNP: The NCBI database of genetic variation. Nucleic Acids Res 29, 308–311. 11125122 10.1093/nar/29.1.308 PMC29783

[53] Chung J , Ushakova A , Doitsidou M , Tzoulis C , Tysnes OB Dalen I , Pedersen KF , Alves G , Maple-Grodem J (2021) The impact of common genetic variants in cognitive decline in the first seven years of Parkinson’s disease: A longitudinal observational study. Neurosci Lett 764, 136243. 34509566 10.1016/j.neulet.2021.136243

[54] Davis MY , Johnson CO , Leverenz JB , Weintraub D , Trojanowski JQ , Chen-Plotkin A , Van Deerlin VM , Quinn JF , Chung KA , Peterson-Hiller AL , Rosenthal LS , Dawson TM , Albert MS , Goldman JG , Stebbins GT , Bernard B , Wszolek ZK , Ross OA , Dickson DW , Eidelberg D , Mattis PJ , Niethammer M , Yearout D , Hu SC , Cholerton BA , Smith M , Mata IF , Montine TJ , Edwards KL , Zabetian CP (2016) Association of GBA mutations and the E326K polymorphism with motor and cognitive progression in Parkinson disease. JAMA Neurol 73, 1217–1224. 27571329 10.1001/jamaneurol.2016.2245 PMC5056861

[55] Skorvanek M , Goldman JG , Jahanshahi M , Marras C , Rektorova I , Schmand B , van Duijn E , Goetz CG , Weintraub D , Stebbins GT , Martinez-Martin P , members of the MDS Rating Scales Review Committee (2018) Global scales for cognitive screening in Parkinson’s disease: Critique and recommendations. Mov Disord 33, 208–218. 29168899 10.1002/mds.27233

[56] Shay KA , Duke LW , Conboy T , Harrell LE , Callaway R , Folks DG (1991) The clinical validity of the Mattis Dementia Rating Scale in staging Alzheimer’s dementia. J Geriatr Psychiatry Neurol 4, 18–25. 2054047 10.1177/089198879100400104

[57] Marson DC , Dymek MP , Duke LW , Harrell LE (1997) Subscale validity of the Mattis Dementia Rating Scale. Arch Clin Neuropsychol 12, 269–275. 14588419

[58] Smith GE , Ivnik RJ , Malec JF , Kokmen E , Tangalos E , Petersen RC (1994) Psychometric properties of the Mattis Dementia Rating Scale. Assessment 1, 123–132. 9465142 10.1177/1073191194001002002

[59] Schmidt KS , Lieto JM , Kiryankova E , Salvucci A (2006) Construct and concurrent validity of the Dementia Rating Scale-2 Alternate Form. J Clin Exp Neuropsychol 28, 646–654. 16723314 10.1080/13803390590949539

[60] Brown GG , Rahill AA , Gorell JM , McDonald C , Brown SJ , Sillanpaa M , Shults C (1999) Validity of the Dementia Rating Scale in assessing cognitive function in Parkinson’s disease. J Geriatr Psychiatry Neurol 12, 180–188. 10616865 10.1177/089198879901200403

[61] Woodard JL , Salthouse TA , Godsall RE , Green RC (1996) Confirmatory factor analysis of the Mattis Dementia Rating Scale in patients with Alzheimer’s disease. Psychol Assess 8, 85–91.

